# Comparative evaluation of six nucleic acid amplification kits for SARS-CoV-2 RNA detection

**DOI:** 10.1186/s12941-021-00443-w

**Published:** 2021-05-22

**Authors:** Shuang Wu, Xiaolu Shi, Qiongcheng Chen, Yixiang Jiang, Le Zuo, Lei Wang, Min Jiang, Yiman Lin, Shisong Fang, Bo Peng, Weihua Wu, Hui Liu, Renli Zhang, Patrick S. L. Kwan, Qinghua Hu

**Affiliations:** Shenzhen Center for Disease Control and Prevention, 8 Longyuan Road, Nanshan District, Shenzhen, 518055 China

**Keywords:** SARS-CoV-2, COVID-19, Nucleic acid detection, Real-time reverse transcriptase PCR (RT-qPCR), Cross-priming isothermal amplification (CPA)

## Abstract

**Background:**

SARS-CoV-2 is a newly emerged coronavirus, causing the coronavirus disease 2019 (COVID-19) outbreak in December, 2019. As drugs and vaccines of COVID-19 remain in development, accurate virus detection plays a crucial role in the current public health crisis. Quantitative real-time reverse transcriptase-polymerase chain reaction (RT-qPCR) kits have been reliably used for detection of SARS-CoV-2 RNA since the beginning of the COVID-19 outbreak, whereas isothermal nucleic acid amplification-based point-of-care automated kits have also been considered as a simpler and rapid alternative. However, as these kits have only been developed and applied clinically within a short timeframe, their clinical performance has not been adequately evaluated to date. We describe a comparative study between a newly developed cross-priming isothermal amplification (CPA) kit (Kit A) and five RT-qPCR kits (Kits B–F) to evaluate their sensitivity, specificity, predictive values and accuracy.

**Methods:**

Fifty-two clinical samples were used including throat swabs (n = 30), nasal swabs (n = 7), nasopharyngeal swabs (n = 7) and sputum specimens (n = 8), comprising confirmed (n = 26) and negative cases (n = 26). SARS-CoV-2 detection was simultaneously performed on each sample using six nucleic acid amplification kits. The sensitivity, specificity, positive/negative predictive values (PPV/NPV) and the accuracy for each kit were assessed using clinical manifestation and molecular diagnoses as the reference standard. Reproducibility for RT-qPCR kits was evaluated in triplicate by three different operators using a SARS-CoV-2 RNA-positive sample. On the basis of the six kits’ evaluation results, CPA kit (Kit A) and two RT-qPCR Kits (Kit B and F) were applied to the SARS-CoV-2 detection in close-contacts of COVID-19 patients.

**Results:**

For Kit A, the sensitivity, specificity, PPV/NPV and accuracy were 100%. Among the five RT-qPCR kits, Kits B, C and F had good agreement with the clinical diagnostic reports (Kappa ≥ 0.75); Kits D and E were less congruent (0.4 ≤ Kappa < 0.75). Differences between all kits were statistically significant (P < 0.001). The reproducibility of RT-qPCR kits was determined using a coefficients of variation (CV) between 0.95% and 2.57%, indicating good reproducibility.

**Conclusions:**

This is the first comparative study to evaluate CPA and RT-qPCR kits’ specificity and sensitivity for SARS-CoV-2 detection, and could serve as a reference for clinical laboratories, thus informing testing protocols amid the rapidly progressing COVID-19 pandemic.

## Introduction

With currently 59,816,510 confirmed cases and 1,410,378 deaths reported globally as of November 26th, 2020, the ongoing coronavirus disease 2019 (COVID-19) pandemic has been rapidly escalating and straining public health systems worldwide [1]. The genome sequence of the newly emerged causative agent, severe acute respiratory syndrome coronavirus 2 (SARS-CoV-2; Wuhan-Hu-1, GenBank accession number MN908947) (http://virological.org/t/novel-2019-coronavirus-genome/319) released for immediate public health support by the Global Initiative on Sharing All Influenza Data (GISAID) has shown that SARS-CoV-2 has 84% nucleotide homology with bat SARS-like coronavirus, 78% homology with the human SARS-CoV, and approximately 50% homology with Middle Eastern respiratory syndrome (MERS)-CoV [1–4]. The most commonly reported symptoms of SARS-CoV-2 infection include fever, dyspnea, leukopenia, lymphocytopenia, and ground-glass opacity commonly observed on chest computed tomography [5]. From an autopsy on the first patient who died from COVID-19, pulmonary manifestations were diffuse alveolar injury and clear membrane formation [6], which were consistent with the manifestations of acute respiratory distress syndrome and similar to the pathological features of SARS- and MERS-CoV infections [7]. In terms of public health, it has become clear that the most effective way to prevent spread of infections is by breaking the chain of transmission through social distancing.

Since the outbreak began, various commercial nucleic acid amplification kits have been developed to provide a simple and reliable means for laboratory detection of SARS-CoV-2. Among kit methodologies, real time reverse transcriptase PCR (RT-qPCR) has been the standard method for laboratory diagnosis of SARS-CoV-2 infection and has already been routinely used for detecting other respiratory viruses. According to China’s latest clinical guideline, the Diagnosis and Treatment Protocol for Novel Coronavirus Pneumonia (Trial Version 7) [8, 9], diagnosis of COVID-19 must be confirmed by RT-qPCR or gene sequencing from respiratory or blood samples, which is a key criterion for hospitalization [10, 11]. Conversely, cross-priming isothermal amplification (CPA) kits that use a closed-tube test have become a simple and fast SARS-CoV-2 test that can be performed by minimally trained personnel at point-of-care without the need for complex equipment [12]. Through specific amplification primers, fluorescent probes and DNA polymerases with high reverse transcriptase activities and chain displacement characteristics, CPA kits could complete the specific amplification process of SARS-CoV-2 fragments at a constant temperature. The fluorescence signal is detected by adaptive instruments and a real-time fluorescence curve is automatically generated.

Owing to high global demand, the development and production of nucleic acid amplification kits by various manufacturers in China have rapidly scaled up, but they have only been applied clinically for a short period of time. Hence, such kits may not have been fully validated for performance or evaluated in clinical application, where both false-negative and false-positive results have been observed [2]. Furthermore, there has been no comparative study between their analytical performance. In this study, our objective was to compare and evaluate six different commercially available nucleic acid amplification kits using clinical diagnositc reports as reference standard to provide the necessary comparative evaluation.

## Materials and methods

### Case definitions

In accordance with the latest guidelines of Diagnosis and Treatment of Novel Coronavirus Pneumonia (Trial Version 7) from the National Health Commission & State Administration of Traditional Chinese Medicine of China (available at http://en.nhc.gov.cn/2020-03/29/c_78469.htm), a suspected case was defined as a person with any of the following epidemiological history plus any two of the following clinical manifestations, or all three clinical manifestations if there was no clear epidemiological history. (1) Epidemiological history: within 14 days prior to the onset of illness, (a) traveled to or took residence, or (b) had contact with individuals who had fever or respiratory symptoms, in Wuhan and geographical proximities or communities where cases had been reported; (c) had contact with any laboratory confirmed cases; (d) was part of a cluster of two or more cases with fever and/or respiratory symptoms. (2) Clinical manifestations: (a) fever and/or respiratory symptoms; (b) defined imaging characteristic of SARS-CoV-2 pneumonia; (c) normal or decreased white blood cell count and/or lymphocyte count in the early stages of illness onset.

Confirmed cases were defined as suspected cases with one of the following etiological evidences: (1) a positive RT-qPCR test for SARS-CoV-2; (2) viral gene sequence highly homologous to SARS-CoV-2; (3) SARS-CoV-2 specific IgM and IgG antibodies detectable in serum, IgG detectable or reaching a titration of at least a four-fold increase during convalescence compared with the acute phase. Negative cases were defined as suspected cases with two consecutive negative nucleic acid tests taken at least 24-h apart and that SARS-CoV-2 specific IgM and IgG antibodies were negative after 7 days from illness onset.

### Clinical samples

Respiratory samples (n = 52) were collected from suspected cases at the Shenzhen Third People’s Hospital and a compulsory quarantine facility in Shenzhen. Specimen types included throat swabs (n = 30), nasal swabs (n = 7), nasopharyngeal swabs (n = 7) and sputum specimens (n = 8), comprising clinical manifestationor PCR-confirmed (n = 26) and -negative (n = 26) cases.

### Nucleic acid amplification

All six kits used the ORF1ab gene and the N gene as targets (ORF1ab only for Kit D) and were performed in accordance with the manufacturer’s instructions (Table 1). Briefly, in the automated CPA kit (Kit A), swab samples were directly applied to cartridges preloaded with reagents for nucleic acid purification, elution buffer and CPA reaction master mix, in which RNA were automatically purified, extracted and amplified. Fluorescence-labeled probes bind specifically to the amplified RNA targets to produce a fluorescent signal detectable by the instrument in real time for the determination of test results. For the RT-qPCR kits (Kits B–F), RNA was manually extracted with a High Pure Viral Nucleic Acid kit (Roche Diagnostics, Mannheim, Germany). For Kit B, the PCR was carried out in a 25 μL reaction volume containing 5 μL isolated DNA as template, 4 μL primer and probe mix (ORF1ab/N) and 4 μL Premix ExTaq. Amplification began with one cycle of reverse transcription at 50℃ for 10 min and a pre-denaturation step at 95 °C for 5 min, followed by 40 cycles of 95 °C for 10 s, 55 °C for 40 s. For Kit C, the PCR was carried out in a 25 μL reaction volume containing 5 μL viral cDNA as template, 19 μL RT-qPCR buffer and 1 μL enzyme mix. Amplification began with one cycle of reverse transcription at 42℃ for 15 min and a pre-denaturation step at 95 °C for 10 min, followed by 40 cycles of 95 °C for 5 s, 60 °C for 45 s. For Kit D, the 30 μL RT-qPCR reaction mixture contained 10 μL viral cDNA as template, 18.5 μL RT-qPCR buffer and 1.5 μL enzyme mix. Amplification began with one cycle of reverse transcription at 50℃ for 20 min and a pre-denaturation step at 95 °C for 10 min, followed by 40 cycles of 95 °C for 15 s, 60 °C for 30 s. For Kit E, the 20 μL RT-qPCR reaction mixture contained 4 μL viral cDNA template, 18.5 μL RT-qPCR buffer and 1.5 μL enzyme mix. Amplification began with one cycle of reverse transcription at 55℃ for 15 min and a pre-denaturation step at 95 °C for 30 min, followed by 45 cycles of 95 °C for 10 s, 60 °C for 30 s. For Kit F, the PCR was carried out in a 25 μL reaction volume containing 5 μL viral cDNA template, 17 μL RT-qPCR Buffer A and 3 μL RT-qPCR Buffer B. Amplification began with one cycle of reverse transcription at 50℃ for 15 min and a pre-denaturation step at 95 °C for 15 min, followed by 45 cycles of 94 °C for 15 s, 55 °C for 45 s. The assays were performed on an ABI 7500 Real Time PCR system (Applied Biosystems, Foster City, CA), Fluorescent signal was detected at the end of the extension step of each cycle. The results were interpreted by CT values generated using specific cut-offs for each kit (Table 1).

**Table 1:** Parameters for each of the six nucleic acid amplification kits evaluated

Kit	Target	Volume (uL)		Results interpretation		LOD (copies/mL)	Ref
		sample	RNA	Positive	Negative		
A	ORF 1ab, N	500	/	Tt ^a^ ≤ 40	No Tt value	1000	Y
B	ORF 1ab, N	/	5	Ct ≤ 38	No Ct value or Ct > 38	1000	N
C	ORF 1ab, N	/	5	Ct ≤ 35	Ct > 38	500	N
D	ORF 1ab	/	10	Ct ≤ 32	FAM: No Ct value VIC/HEX: Ct ≤ 32	100	Y
E	ORF 1ab, N	/	4	Ct < 38	No Ct value or Ct = 45	300	N
F	ORF 1ab, N	/	5	Ct ≤ 40	No Ct value or Ct > 40	500	Y

^a^Tt value is unique to the CPA kit (Kit A) and represents the time when fluorescence value exceeds the threshold line

### Analytical performance of six nucleic acid amplification kits

Analytical sensitivity and specificity, the positive and negative predictive values (PPV and NPV) and the accuracy for each kit were assessed using clinical diagnostic reports as the reference standard.

### Reproducibility of RT-qPCR kits

Reproducibility for the RT-qPCR kits was evaluated using a positive sample verified by all kits, measured in triplicate by three different operators. The means, standard deviations (SD) and the coefficients of variation (CV) were calculated.

### Statistical analysis

Statistical analyses were performed using SPSS software version 21.0 (IBM Corp., Armonk, NY). Confidence intervals (95%) were computed using the Wilson Score method. The agreement between nucleic acid amplification kits and standard diagnosis methods were compared using the McNemar-Bowker test.

### Clinical application

Additional throat swabs (n = 200) from close contacts of confirmed cases were obtained from the compulsory quarantine facility to further compare Kit A with Kits B and F (approved and authorized by the National Medical Products Administration for clinical use). Agreement between three kits was assessed.

## Results

### Performance characteristics of six kits

From the clinical samples tested (n = 52), which comprised both confirmed (n = 26) and negative (n = 26) cases by clinical manifestation and standard RT-qPCR methods, the analytical performances from the six kits were evaluated using previous diagnosis as the reference standard (Table 2). The analytical sensitivity and NPV of all six kits were 100%. The analytical specificity and PPV of the CPA kit (Kit A) were both 100% (95% CI, 87.1–100.0), and the kappa value was 1.0 (*P* < 0.001). For RT-qPCR kits (Kits B–F), Kits B, C and F showed high agreement with standard diagnosis techniques (Kappa ≥ 0.75, *P* < 0.001), while Kits D and E were generally consistent with standard diagnosis methods (0.4 ≤ Kappa < 0.75, *P* < 0.001). Differences observed between the six kits were statistically significant (*P* < 0.001).

**Table 2:** Performance characteristics of six nucleic acid amplification kits for the detection of SARS-CoV-2 using clinical diagnostic reports as the reference standard

	No. of RT-qPCR result	No. of clinical diagnostic report		Sensitivity	Specificity	PPV	NPV	Diagnostic accuracy	Kappa
		Positive	Negative	(95% CI)	(95% CI)	(95% CI)	(95% CI)	(95% CI)	
A	Positive	26	0	100 (87.1–100)	100 (87.1–100)	100 (87.1–100)	100 (87.1–100)	100	1
	Negative	0	26					(93.1–100)	(p < 0.001)
B	Positive	23	0	100 (85.7–100)	89.7 (73.6–96.4)	88.5 (71.0–96.0)	100 (87.1–100)	94.2	0.885
	Negative	3	26					(84.4–98.0)	(p < 0.001)
C	Positive	25	0	100 (86.7–100)	96.3 (81.7–99.3)	96.2 (81.1–99.3)	100 (87.1–100)	98.1	0.962
	Negative	1	26					(89.9–99.7)	(p < 0.001)
D	Positive	18	0	100 (82.4–100)	76.5 (60.0–87.6)	69.2 (50.0–83.5)	100 (87.1–100)	84.6	0.692
	Negative	8	26					(72.5–92.0)	(p < 0.001)
E	Positive	20	0	100 (83.9–100)	81.3 (64.7–91.1)	76.9 (57.6–89.0)	100 (87.1–100)	88.5	0.769
	Negative	6	26					(77.0–94.6)	(p < 0.001)
F	Positive	24	0	100 (86.2–100)	92.9 (77.4–98.0)	92.3 (75.9–97.9)	100 (87.1–100)	96.15	0.923
	Negative	2	26					(87.0–98.9)	(p < 0.001)

### Reproducibility of RT-qPCR kits

The SDs and CVs of the fifty-two clinical samples for the five RT-qPCR kits were between 0.35 and 0.87, and 0.95 and 2.57%, respectively, indicating high reproducibility (Table 3).

**Table 3:** Reproducibility of five RT-qPCR kits (Kits B–F)

Kit	1	2	3	4	5	6	7	8	9	Mean(℃)	SD	CV(%)
B	34.56	33.80	34.79	35.49	34.37	33.99	35.32	33.43	33.72	34.39	0.72	2.09
C	31.72	30.38	31.90	31.61	32.41	31.85	30.35	32.31	31.61	31.57	0.74	2.34
D	34.54	34.34	34.36	35.77	33.19	33.45	33.88	33.26	33.06	33.98	0.87	2.57
E	32.74	31.78	33.03	32.55	31.99	32.66	32.61	32.99	33.91	32.70	0.62	1.89
F	36.15	36.54	36.34	36.87	37.22	36.81	36.29	36.61	36.95	36.64	0.35	0.95

### Clinical application

Among throat swab samples from close contacts (n = 200), one sample from a confirmed case tested positive for SARS-CoV-2 with all three (Kits A, B and F) kits, indicating total agreement between the three kits.

## Discussion

COVID-19 caused by SARS-CoV-2 has now become a global pandemic and caused substantial mortality and morbidity internationally due to rapid disease propagation. The rapid and accurate identification of pathogenic virus is of great significance to the selection of appropriate treatment, saving of lives and prevention of infectious disease. To date, China has rapidly developed and approved more than 10 SARS-CoV-2 detection methods [10]. In this study, the analytical performance of six kits, including a CPA kit and five RT-qPCR kits were compared and evaluated using clinical diagnostic reports as the reference standard. RT-qPCR tests are often used in the detection pathogenic viruses in respiratory secretions and for conclusive diagnosis of COVID-19 [13]. It is a robust and reliable technique and has been widely used in laboratory tests and scientific experiments. However, RT-qPCR also has some drawbacks. For example, it could take up to 4 h or longer from initiation, including nucleic acid extraction, amplification reactions and analysis, to reporting results. Therefore, shortening the process of nucleic acid extraction and amplification time would be a useful improvement for RT-qPCR. Our evaluation of RT-qPCR kits showed that Kits B, C and F had good agreement with standard diagnosis, while Kits D and E were less consistent, all of which gave false negative results. According to an estimate, the false negative rate (FNR) of RT-qPCR may be as high as 30%–50% in real COVID-19 cases. Several factors have been proposed to explain the inconsistency or high FNR [13]. For example, this could be caused by patients who were asymptomatic or only displayed mild symptoms after infection, since the viral load of them are usually very low. The viral load sampled from patients may also vary, depending on disease progression or sample quality, which could be one of the main reasons why clinical patients can repeatedly test negative but then test positive at a later date. Therefore, nucleic acid testing should be carried out by repeated collection of specimens for suspected cases. Furthermore, primers for the target genes ORF1ab and N can be affected by virus genetic variation [13]. We have also found that sampling method, sampling depth, time of the swab stayed on the site and number of times it was rotated during sampling are related to the sample quality, which affects the results of RT-qPCR. In essence, a negative result for the first time should not be considered a confirmed laboratory diagnosis, especially in areas with high disease incidence. In contrast to RT-qPCR, CPA is a super-sensitive nucleic acid amplification method that can usually detect small amounts of RNA template with minimal sample preparation [10]. It is also often fully automated and could be used by personnel in the field or at point-of-care with minimal training, so the biosecurity risks are lower. From our evaluation, the CPA kit (Kit A) showed excellent analytical performance, with 100% sensitivity, specificity and accuracy. The main drawback of the CPA kit is a low-throughput device, which could only process two samples at once; another minor drawback in its methodology is that specific high temperatures are required.

Our current comparative evaluation also had certain limitations. Owing to the small number of confirmed cases in our study, there was a lack of sample diversity in our tests, coupled with a limited number of reagent batches used in our assays. Therefore, the limited sample size may not accurately reflect true analytical performance. During this outbreak, samples may also have been repeatedly frozen and thawed, which may have had an impact on the results. If possible, the clinical specimen sample size should be increased for more robust evaluation of the nucleic acid amplification kits for SARS-CoV-2 detection.

## Conclusion

Each analysis method has its unique advantages and inevitable disadvantages. CPA has advantages of fast amplification, simple operation and convenient detection; it is an ideal candidate for the development of portable molecular diagnostics, which is suitable for field screening detection and point-of-care testing. Conversely, RT-qPCR kits are highly sensitive and specific with high-throughput capabilities, making these a more common choice for a laboratory setting. For COVID-19, we still need to develop more effective and practical methods to overcome the shortcomings of existing methods, while weighing the advantages and disadvantages of the various detection methods, to obtain the most economical and efficient choice.

## Data Availability

The datasets generated for this study are available on request to the corresponding author.
